# Assisted Reproductive Technology Outcomes in Women With Heart Disease

**DOI:** 10.3389/fcvm.2022.842556

**Published:** 2022-04-11

**Authors:** Mary M. Quien, Anaïs Hausvater, Susan M. Maxwell, Catherine R. Weinberg

**Affiliations:** ^1^Bridgeport Hospital, Yale New Haven Health, Bridgeport, CT, United States; ^2^Leon H. Carney Division of Cardiology, New York University Langone Health, New York, NY, United States; ^3^Northwell Health, New York, NY, United States

**Keywords:** assisted reproductive technology, *in-vitro* fertilization, congenital heart disease, modified WHO classification, maternal cardiac risk, infertility and heart disease

## Abstract

**Background:**

Women with infertility and heart disease (HD) are increasingly seeking assisted reproductive technology (ART). There is only one other study that examines the safety profile of ART in this population. This study aims to evaluate the cardiac, reproductive, and obstetric outcomes of ART in women with HD.

**Methods:**

We conducted a retrospective case-control study of women with underlying congenital or acquired HD who underwent ART at a single University fertility center from 1/2010–3/2019. Women undergoing *in-vitro* fertilization (IVF), oocyte cryopreservation (OC) or embryo banking (EB) with HD were included. Cases were matched 3:1 with age-, cycle type- and cycle start date- matched controls without HD. Outcomes included cardiovascular (CV), reproductive, and obstetric complications during or following ART.

**Results:**

Twenty women with HD were included. 15 (75%) had congenital HD, 1 (5%) had valvular disease, 1 (5%) had acquired cardiomyopathy, and 3 (15%) had arrhythmias. 90% were New York Heart Association class I. 55% of HD cases were modified WHO (mWHO) risk classification 1-2, 40% were mWHO 2-3 or 3, 5% were mWHO 4. Cases underwent 25 IVF, 5 OC, and 5 EB cycles and were compared with 79 controls who underwent 174 cycles. No CV complications or deaths occurred amongst cases following ART or pregnancy. There was no difference in risk of ART or obstetric outcomes amongst cases versus controls.

**Conclusion:**

For women with HD in this small, low -risk cohort, ART posed few risks that were similar in frequency to healthy controls.

## Introduction

Women with cardiac disease seeking pregnancy are at increased of risk of ventricular dysfunction, arrhythmias, pre-eclampsia, *caesarean* sections, and postpartum hemorrhage (1). One in four women with cardiac disease in pregnancy are hospitalized during their pregnancy, and cardiovascular disease is the biggest indirect cause of maternal death worldwide, with an attributable rate of two deaths per 100,000 (2, 3). To better understand maternal risk, the European Society of Cardiology currently recommends utilizing the modified WHO (mWHO) classification to assess maternal risk of cardiac complications during pregnancy and recognizes that the Cardiac Disease in Pregnancy (CAPREG) and Zwangerschap bij Aangeboren HARtAfwijkingen I (ZAHARA) scoring systems can be used to further estimate risk (3, 4). Women with heart disease at high risk of pregnancy related complications based on the above scoring systems are thus increasingly looking to assisted reproductive technology (ART) as a means to preserve their fertility (5). ART can be used to create embryos and allow high risk patients to have children through the use of a gestational carrier.

Assisted reproductive technology (ART) is becoming widely implemented worldwide. In the United States alone in 2016, there were over 86,000 ART cycles, which resulted in ~2% of all live births (6). Although ART is becoming a more popular fertility treatment option, there are known risks involved with the process. Studies have shown that women undergoing *in-vitro* fertilization (IVF) have an increased incidence of complications, such as eclampsia, postpartum hemorrhage, and thromboembolic disease (7). In one study, investigators found that venous thromboembolism occurred in 4.2 per 1,000 women after IVF compared with 2.5 per 1,000 in women with natural pregnancies (8). One meta-analysis also found that IVF was associated with acute changes in hemodynamic parameters, with the most profound changes occurring around the days of embryonic implantation when GnRH agonist protocols are used (9). Furthermore, to obtain oocytes for ART, women must undergo controlled ovarian hyperstimulation, which can be complicated by ovarian hyperstimulation syndrome (OHSS). This phenomenon can potentially cause fluid shifts, electrolyte abnormalities, ascites, and in rare instances, pleural effusions. Oocyte retrieval also poses the risk of bleeding and anesthesia related risks (10). These complications can lead to life threatening situations, especially in women with compromised cardiac function.

While one study by Dayan et al. (11) has looked at the pregnancy outcomes and complications in women with cardiac diseases undergoing IVF, the safety of ART procedures in women with heart disease remains sparce. Thus, the current study aims to evaluate the cardiac and obstetric outcomes of ART in women with heart disease (HD) as compared to the general population.

## Methods

We conducted a retrospective case-control study of women aged 18 years and older with underlying cardiovascular disease who underwent ART at a single university fertility center between January 2010 and March 2019. Women undergoing IVF, oocyte cryopreservation (OC) or embryo banking (EB) with heart disease were included. Cases were matched 3:1 with age-, cycle type- and cycle start date- matched controls without heart disease. This study was approved by the Institutional Review Board of New York University Langone Health. Consent was obtained at the time of treatment.

Patients were classified as having either acquired or congenital heart disease (CHD). Acquired heart disease was further categorized as valvular disease, arrhythmia, cardiomyopathy, and ischemic heart disease. Cases were categorized based on maternal risk using the mWHO criteria, in which WHO class I is associated with very low risk of maternal cardiac events (2.5–5%), class II are low-moderate risk (5.7–10.5%), WHO II–III are moderate risk (10–19%), WHO III are at high risk (19–27%), and WHO IV (40–100%) in which women should be advised against pregnancy (4).

Primary outcomes included cardiovascular (CV) complications during or following ART (arrhythmias, heart failure, hypotension, thrombosis, or CV death), reproductive complications, and obstetric complications. Secondary outcomes included obstetric outcomes and neonatal outcomes and complications. Statistical analysis was conducted using SPSS. Two tailed paired *t*-tests were conducted to calculate statistical significance.

## Results

Twenty cases underwent a total of 25 IVF cycles, 5 oocyte cryopreservation cycles, and 5 embryo banking cycles, whereas 79 controls had total of 174. Average age of cases was 35.8 ± 5.3 years, which was similar to that of controls with an average age of 34.7 ± 4.9 years. The majority of cases (*N* = 17, 85%) and controls (*N* = 50, 63%) were Caucasian. There was no significant difference in co-morbidities between the two groups. The most common co-morbidity among cases was diabetes mellitus (*N* = 2, 10%). The most common co-morbidity among controls was polycystic ovarian syndrome (*N* = 8, 10%) (Table 1). 11 (55%) cases and 39 (49%) controls were nulliparous at the start of ART.

**Table 1:** Baseline characteristics of women with HD and controls.

	Women with HD^*^(*N =* 20)	**Control patients (*N =* 79)**	***P*-Value**
Age (years), mean (STD)	35.8 (±5.3)	34.7 (±4.9)	*P =* 0.43
**Race**			
Caucasian	17 (85%)	50 (63%)	
Asian/Indian	2 (10%)	12 (15%)	
African	0 (0%)	1(1%)	
Other	0 (0%)	4 (5%)	
Unknown	1 (5%)	12 (15%)	
**Ethnicity**			
Hispanic	0 (0%)	3 (4%)	
**Comorbidities**			
Hypertension (%)	1 (5%)	1 (1%)	*P =* 0.36
Diabetes Mellitus (%)	2 (10%)	1 (1%)	*P =* 0.10
Stroke (%)	1 (5%)	0 (0%)	*P =* 0.20
Chronic Kidney Disease (%)	1 (5%)	0 (0%)	*P =* 0.20
Hypothyroidism (%)	1 (5%)	7 (9%)	*P =* 0.69
Obesity (BMI > 30) (%)	0 (0%)	2 (3%)	*P =* 1
Polycystic Ovarian Syndrome (%)	1 (5%)	8 (10%)	*P =* 0.68

* *HD, heart disease*.

**Figure 1:**
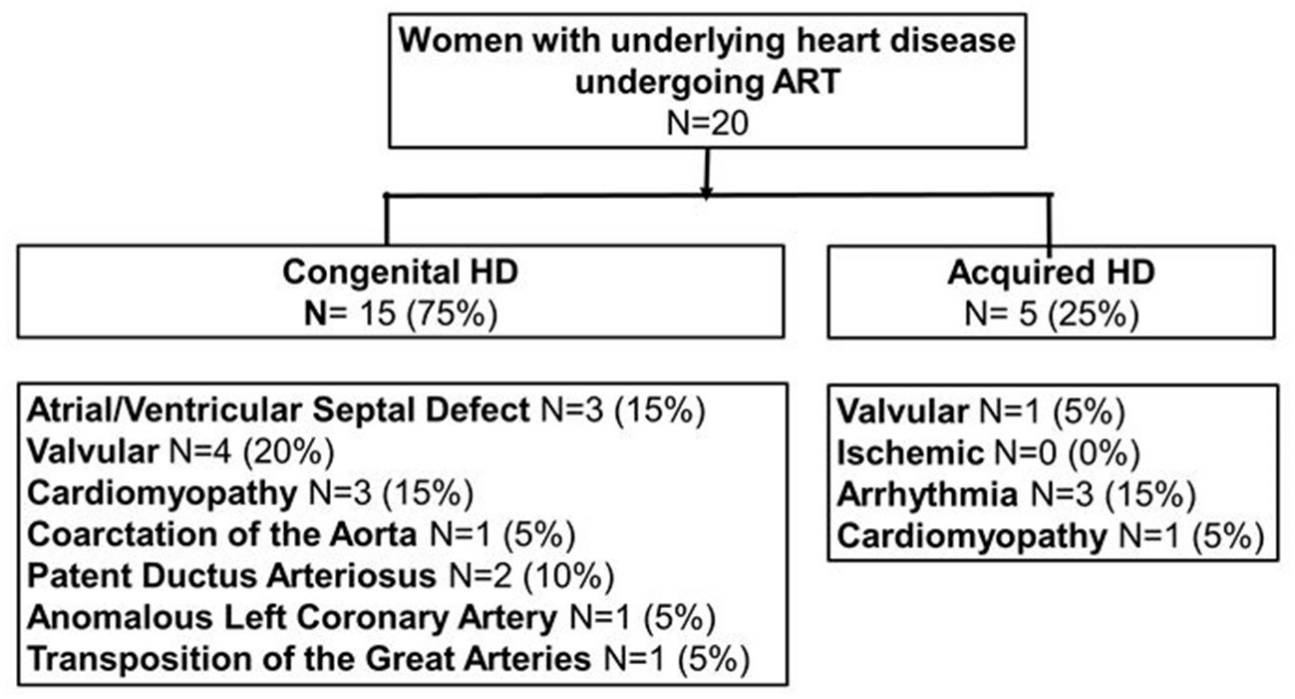
Classification of subjects with heart disease into acquired (*N* = 5) and congenital heart disease (*N* = 15). ART, assisted reproductive technology; HD, heart disease.

Of 20 cases, 15 (75%) had CHD, 1 (5%) had acquired valvular disease, 1 (5%) had acquired cardiomyopathy, and 3 (15%) had arrhythmias. Further breakdown of the type of HD is shown in Figure 1. The majority of cases (*N* = 6, 30%) were mWHO class II. 5 (25%) cases were mWHO class I, 3 (15%) were mWHO class II-III, 5 (25%) were mWHO class III, and 1 (5%) was mWHO class IV. Ninety percentage of cases were New York Heart Association (NYHA) class I at the time of ART. To proceed with ART therapy, all patients had to be candidates for outpatient sedation.

The mWHO class III patients consisted of a case of non-compaction cardiomyopathy with a normal ejection fraction (EF), a case of anomalous left coronary artery who underwent bypass surgery, a case of dextro-transposition of the great arteries who underwent the Blalock-Hanlon procedure and arterial switch procedure with a mechanical aortic valve, a case of dilated cardiomyopathy with a reduce EF of 35–40%, and a case of hypertrophic cardiomyopathy who had undergone myomectomy and eventually heart transplant. The mWHO class IV patient was a case of peripartum cardiomyopathy with an EF of 25–30%. Three cycles (one mWHO class IV and 2 mWHO class III) utilized gestational carriers.

Among cases, there were no CV complications during or after ART cycle. Among controls, 1 (1%) cycle was complicated by stroke after ART cycle and 1 (1%) cycle was complicated by hypotension during the ART cycle. No death occurred in either group as a result of ART. The most common ART complication in both groups was ovarian hyperstimulation syndrome (OHSS) with 1 (3%) cycle in cases and 8 (5%) cycles in controls. The incidence of cardiovascular and ART complications was similar amongst cases vs. controls (Table 2).

**Table 2:** Cardiovascular and ART complications of women with HD and controls.

	ART^*^cycles in women with HD^†^(*N =* 35)	**Control ART cycles in controls (*N =* 174)**
**Cardiac outcome**		
Arrhythmia (%)	0 (0%)	0 (0%)
Thrombosis (%)	0 (0%)	1 (1%)
Hypotension (%)	0 (0%)	1 (1%)
Heart failure (%)	0 (0%)	0 (0%)
Cardiac death (%)	0 (0%)	0 (0%)
**ART outcome**		
Bleeding during ovum retrieval (%)	0 (0%)	0 (0%)
OHSS^‡^ (%)	1 (3%)	8 (5%)
Syncope (%)	1 (3%)	1 (1%)

* *ART, assisted reproductive technology*.

† *HD, heart disease*.

‡ *OHSS, ovarian hyperstimulation syndrome*.

For obstetric outcomes, 8 (32%) IVF cycles resulted in pregnancy in cases compared to 69 (46%) IVF cycles among controls. For those who became pregnant among cases, 6 (24%) cycles resulted in single gestation, 0 in multiple gestation, 1 (4%) in spontaneous abortion, and 1 (4%) in ectopic pregnancy. There were no pre-term pregnancies among cases. For controls, 47 (31%) cycles resulted in single gestation, 4 (3%) in multiple gestation, and 18 (12%) in spontaneous abortion. There were no ectopic pregnancies. 8 (12%) of control pregnancies were pre-term.

Among cases, 1 (13%) pregnancy was complicated by pre-eclampsia, and 1 (13%) was complicated by antepartum bleeding. In controls, 9 (13%) pregnancies were complicated by pre-eclampsia, 4 (6%) by gestational diabetes, and 1 (1%) by postpartum hemorrhage. No death occurred in either group as a result of pregnancy. The incidence of obstetric outcomes and complications were similar amongst cases vs. controls (Table 3). In addition, there were no neonatal complications amongst the cases. There was one incidence of a congenital abnormality amongst a control.

**Table 3:** Obstetric outcomes and complications of women with HD and controls.

	Pregnancy in women with HD^*^(*N =* 8)	**Control pregnancies (*N =* 79)**
**Obstetric outcome**		
Single gestation (%)	6 (75%)	47 (68%)
Multiple gestation (%)	0 (0%)	4 (6%)
Spontaneous abortion (%)	1 (13%)	18 (26%)
Ectopic pregnancy (%)	1 (13%)	0 (0%)
**Obstetric complications**		
Maternal non-cardiac death (%)	0 (0%)	0 (0%)
Antepartum hemorrhage (%)	1 (13%)	0 (0%)
Post-partum hemorrhage (%)	0 (0%)	1 (1%)
Pre-eclampsia (%)	1 (13%)	9 (13%)
Gestational diabetes (%)	0 (0%)	4 (6%)

* *HD, heart disease*.

## Discussion

This study demonstrates that in our cohort of women with acquired or underlying congenital HD, ART has a safety profile that is similar in frequency to healthy controls with no history of cardiac disease. Similar to studies that examined ART in the general population, OHSS was a significant ART complication (12). The British Royal College of Obstetricians and Gynecologists cites an incidence for mild OHSS of about one in every three cycles of IVF and an incidence for moderate or severe forms ranging from 3.1 to 8% of cycles (13). This is similar to the incidence in our cohort. However, a study by Dayan et al., which explored a similar population to ours, found an increased incidence of OHSS (18% vs. 1%) when compared to a population-based study (11).

For pregnancy and neonatal outcomes, our cohort demonstrated lower incidence of complications than other studies. The aforementioned study by Dayan et al., for example, noted an increased incidence of maternal cardiovascular complications (27% vs. 13%), and neonatal complications (45% vs. 20%) in women with HD when compared to a population-based study (11). A systematic review of 50 cohort studies by Qin et al. also demonstrated significantly increased maternal risk outcomes associated with ART that were not seen in our population, such as placenta previa (271% increase) and perinatal mortality (64% increase) (14).

Several factors can possibly account for these differences. First, this study documented a greater number of ART cycles, and the aforementioned Dayan et al. study documented only self-reported complications. That study also did not have a control group. Moreover, ART is a relatively safe practice. In one study of 23,827 transvaginal oocyte retrieval procedures, <1% of patients suffered complications with anesthesia complications comprising only 0.06% (15). Our sample size may have been too small to demonstrate the increased risk of ART complications.

An important factor also lies in the differences between the studied cohorts. The vast majority of our cases were NYHA functional class I and had to be considered low risk enough to receive outpatient sedation for the ART procedure. The Dayan et al. study only investigated cases that resulted in pregnancy rather than all cycles. Pregnancies in women with heart disease have previously been shown to be associated with cardiac events, which could account for that study's increased incidence in cardiac complications (16, 17). Also, there were no cases of multiple gestations in the HD group in our study, a higher risk pregnancy, which may have led to underestimation of risk. Furthermore, two patients of the HD group were deemed to be high-risk and used gestational carriers, which offset the risk of pregnancy complications. Due to this risk stratification, our lower risk cohort may not be representative of majority high risk cohorts.

Overall reduction of complications seen in women with HD undergoing ART can also be attributed to both improved care of patients with HD and advancements in ART. Awareness of the medical complexity of patients with CHD, for example, has led to specialty clinics and specific guidelines for transition of care from childhood into adulthood (18, 19). Different aspects of ART treatment can also be adjusted for patients at increased risk. The IVF protocol, for example, can be altered to a GNRH antagonist-based protocol to reduce the risk of OHSS while maintaining a similar pregnancy rate (20). Physicians have also identified potentially hemodynamically compromising investigations conducted during fertility investigations, such as hysterosalpingograms. While this test is benign in most patients, pain and cervical manipulation can result in a vagal response, which can be potentially dangerous in women with pulmonary hypertension or a Fontan repair (21). This complication can be prevented by implementing cardiovascular monitoring and providing adequate pain relief with either a paracervical block or systemic opioids (22, 23). If patients remain at high-risk despite precautions, physicians are aware to recommend gestational carriers.

Advancements in embryo culture and cryopreservation techniques and the establishment of embryo transfer guidelines have also reduced the risk of OHSS, multiple gestations, and their associated complications (24). The reduction in the number of embryos transferred and the use of pre-implementation genetic testing for aneuploidy may account for the absence of multiple gestations seen in our cases. The success of these techniques speaks toward the possibility for a standardized ART protocol for women with HD to ensure reduction of cardiovascular and obstetric complications.

This study is limited by its retrospective design, single center analysis, and small sample size. Our study also did not include any patients with ischemic heart disease, a subgroup of women that deserves further study. Importantly, our cohort did not include any patients of African American or Hispanic ethnicity so the results cannot be extended to these populations. Additionally, our study is limited in the timeline of our analysis. We do not have data on follow-up beyond the immediate post-partum period, so findings do not account for the possibility of long-term cardiac complications as a result of ART (25).

This study found that for women with primarily low risk HD, ART does not pose any more cardiac, reproductive, or obstetric risk when compared to healthy age- and cycle- matched controls. Given our limited scope, further studies with a more ethnically diverse cohort are needed to confirm the short-term and long-term safety of ART in patients with various types of cardiac disease and to evaluate the success rate of ART in this population. With this information, physicians referring women with cardiac disease for ART may be better suited to counsel their patients on fertility treatment options and their procedural risks.

## Data Availability Statement

The original contributions presented in the study are included in the article/Supplementary Material, further inquiries can be directed to the corresponding author/s.

## Ethics Statement

The studies involving human participants were reviewed and approved by New York University Langone Health. The patients/participants provided their written informed consent to participate in this study. Written informed consent was obtained from the individual(s) for the publication of any potentially identifiable images or data included in this article.

## Author Contributions

SM and CW contributed to conception and design of the study. SM and MQ collected and organized the data. AH performed the statistical analysis. MQ and AH wrote sections of the manuscript. All authors contributed to manuscript revision, read, and approved the submitted version.

## Conflict of Interest

The authors declare that the research was conducted in the absence of any commercial or financial relationships that could be construed as a potential conflict of interest.

## Publisher's Note

All claims expressed in this article are solely those of the authors and do not necessarily represent those of their affiliated organizations, or those of the publisher, the editors and the reviewers. Any product that may be evaluated in this article, or claim that may be made by its manufacturer, is not guaranteed or endorsed by the publisher.
